# Metabolomics Reveals Reduction of Metabolic Oxidation in Women with Polycystic Ovary Syndrome after Pioglitazone-Flutamide-Metformin Polytherapy

**DOI:** 10.1371/journal.pone.0029052

**Published:** 2011-12-16

**Authors:** Maria Vinaixa, Miguel Angel Rodriguez, Sara Samino, Marta Díaz, Antoni Beltran, Roger Mallol, Cinta Bladé, Lourdes Ibañez, Xavier Correig, Oscar Yanes

**Affiliations:** 1 Metabolomics Platform of the Spanish Biomedical Research Center in Diabetes and Associated Metabolic Disorders, Rovira i Virgili University, Tarragona, Spain; 2 Institut d'Investigació Sanitària Pere Virgili, Reus, Spain; 3 CIBER de Diabetes y Enfermedades Metabólicas Asociadas, Barcelona, Spain; 4 Endocrinology Unit, Hospital Sant Joan de Déu-Universitat de Barcelona, Esplugues de Llobregat, Spain; Ohio State University, United States of America

## Abstract

Polycystic ovary syndrome (PCOS) is a variable disorder characterized by a broad spectrum of anomalies, including hyperandrogenemia, insulin resistance, dyslipidemia, body adiposity, low-grade inflammation and increased cardiovascular disease risks. Recently, a new polytherapy consisting of low-dose flutamide, metformin and pioglitazone in combination with an estro-progestagen resulted in the regulation of endocrine clinical markers in young and non-obese PCOS women. However, the metabolic processes involved in this phenotypic amelioration remain unidentified. In this work, we used NMR and MS-based untargeted metabolomics to study serum samples of young non-obese PCOS women prior to and at the end of a 30 months polytherapy receiving low-dose flutamide, metformin and pioglitazone in combination with an estro-progestagen. Our results reveal that the treatment decreased the levels of oxidized LDL particles in serum, as well as downstream metabolic oxidation products of LDL particles such as 9- and 13-HODE, azelaic acid and glutaric acid. In contrast, the radiuses of small dense LDL and large HDL particles were substantially increased after the treatment. Clinical and endocrine-metabolic markers were also monitored, showing that the level of HDL cholesterol was increased after the treatment, whereas the level of androgens and the carotid intima-media thickness were reduced. Significantly, the abundance of azelaic acid and the carotid intima-media thickness resulted in a high degree of correlation. Altogether, our results reveal that this new polytherapy markedly reverts the oxidant status of untreated PCOS women, and potentially improves the pro-atherosclerosis condition in these patients.

## Introduction

Hyperandrogenemia, insulin resistance, a state of low-grade inflammation, body adiposity and a pro-atherogenic lipid profile are usually present in adolescents and young women with polycystic ovary syndrome (PCOS) [1], [2], [3]. Previous reports have evidenced that the PCOS phenotype can also concur with primary alterations of lipid metabolism involving increased levels of oxidized LDL (oxLDL) particles [4], [5], [6]. Accordingly, PCOS may accelerate the development of a cardiovascular-risk profile even in the absence of clinical signs of atherosclerosis. Whether this adverse pro-atherogenic profile and the enhanced oxidant status may increase the risk for cardiovascular disease remains unclear [7], [8], [9].

Most current pharmacological therapies are addressed to ameliorate menstrual irregularities and cosmetic issues. There is a clear need, however, for treatments that also improve endocrine-metabolic markers associated with this disorder. Low-dose flutamide (Flu, a pure androgen receptor blocker) and metformin (Met, an insulin sensitizer) in combination with an estro-progestagen, is a polytherapy that reduces total and abdominal fat, decreases the lean mass deficit, and attenuates the abnormal pattern of adipokines in young and non-obese PCOS women [1]. Recently, it has been reported that the addition of low-dose pioglitazone (Pio, a PPARγ agonist) to the aforementioned polytherapy confers further reductions of visceral fat and carotid intima-media thickness (IMT), and increases further circulating high molecular-weight (HMW) adiponectin [10], [11], [12], [13]. Although the phenotypic evidences demonstrate endocrine-metabolic improvements in a wide spectrum of long-term health markers, the molecular mechanisms underlying such polytherapy remain to be elucidated.

An approach to explore the metabolic changes in PCOS women caused by this new treatment is metabolomics, defined as the metabolic complement of functional genomics. Metabolomics enables the characterization of endogenous small molecules that serve as direct signatures of biochemical activity and therefore are easier to correlate with phenotype. With the ultimate goal of the comprehensive metabolome coverage, there is an overriding need for analytical methodologies able to produce comprehensive metabolite profiles from complex biological samples. However, due to the huge physico-chemical diversity of metabolites, there is not a unique analytical technology able to cope with the whole metabolome. Mass spectrometry (both GC-MS and LC-MS) and NMR have demonstrated to be complementary analytical technologies in metabolomics-based studies [14], [15], [16], allowing to expand the number of metabolites that can be comprehensively covered in an untargeted experiment. Advantages and disadvantages of each analytical technique and their current status in the field of metabolomics are excellently reviewed elsewhere [17], [18], [19], [20].

Here we present a longitudinal study to reveal the mechanism of action of the low-dose Pio/Flu/Met polytherapy using metabolomics. We analyzed using LC-MS, GC-MS and NMR-based metabolomics serum samples of twelve young, non-obese women diagnosed with PCOS, prior to and at the end of a 30 months treatment with low-dose Pio/Flu/Met in combination with an oral estro-progestagen. Our results show that at the end of the treatment there are marked changes in the size of different lipoprotein particles, in conjunction with downstream metabolic oxidation products of LDL particles.

## Methods

### Participants

The study population consisted of twelve young, non-obese women (age, 19.6±0.4 yr; BMI, 22.3±0.9 Kg/m2) diagnosed with PCOS, participating in a randomized study [11], and whose metabolomic profiles were assessed at baseline and after 30 months of treatment. Over 30 months, all women received the same therapy for 24/28 days: pioglitazone (7.5 mg/d) at breakfast, and metformin (850 mg/d), flutamide (62.5 mg/d) and a contraceptive (ethinylestradiol 20 µg/d plus drospirenone 3 mg/d; Yasminelle, Schering) at dinner time.

The inclusion criteria were: 1) hyperinsulinemia on a standard 2-h oral glucose tolerance test, defined as peak insulin levels >150 U/mL and/or mean serum insulin >84 µU/mL; 2) ovarian hyperandrogenism, as defined by each of the following symptoms: hirsutism (Ferriman & Gallwey score >8); amenorrhea (no menses for >3 months) or oligomenorrhea (duration of cycles >45 days); biochemical androgen excess, as judged by circulating androstenedione, total testosterone or free androgen index [FAI, testosterone×100/sex hormone-binding globulin (SHBG)]; 17-OH-progesterone hyperresponse (>160 ng/dL) to GnRH agonist stimulation (leuprolide acetate 500 µg subcutaneously) [10].

The main exclusion criteria were: BMI <17 Kg/m2 or >29 Kg/m2; evidence of thyroid dysfunction; Cushing syndrome or hyperprolactinemia; glucose intolerance; personal history of diabetes mellitus; late-onset adrenal hyperplasia; abnormal liver or kidney function; abnormal blood counts or serum electrolytes; and treatment with an oral contraceptive or another medication known to affect gonadal or adrenal function, carbohydrate or lipid metabolism.

Patients were selected based on available serum samples pre- and post-treatment. PCOS patients receiving placebo for 30 months did not meet ethical requirements and were not included in the clinical study. Blood sampling was performed at baseline and after 30 months on a cyclic off-treatment day (4/28 days). For metabolomics analysis, serum samples (600 µL) were obtained allowing plasma to clot at room temperature for 30 min. After centrifugation at 4°C at 10,000 g for 10 min, samples were maintained at −80°C until further analysis.

### Experimental Procedures

Clinical and endocrine-metabolic variables, carotid IMT, body composition [by dual-energy X-ray absorptiometry (DXA)] and abdominal fat partitioning [by magnetic resonance imaging (MRI)] were assessed prior to and at the end of the treatment as previously described [9], [10], [11], [12]. Sampling was performed in the follicular phase of the cycle, or after 2 months of amenorrhea. Hirsutism was graded according to the Ferriman and Gallwey score [11]. Fasting blood glucose, serum insulin, LDL- and HDL-cholesterol, sex hormone-binding globulin (SHBG), testosterone, androstenedione and dehydroepiandrosterone-sulfate (DHEAS), carotid IMT, body composition and abdominal fat partitioning were measured as previously described [10], [11], [12], [13].

Untargeted metabolomics analysis on serum samples was performed using three different analytical platforms: NMR, GC/MS and LC/ESI-MS TOF; each serum sample was split into three aliquots and run in parallel using the three analytical platforms.

For the NMR measurement 300 µL of serum were mixed with 300 µL of phosphate buffer (0.75 mM Na_2_HPO_4_ adjusted at pH 7.4, and 20% D_2_O to provide the field frequency lock). The final solution was transferred to a 5 mm NMR tube and kept refrigerated at 4°C in the autosampler until the analysis. ^1^H-NMR spectra were recorded at 310 K on a Bruker Avance III 600 spectrometer® operating at a proton frequency of 600.20 MHz using a 5 mm CPTCI triple resonance (^1^H, ^13^C, ^31^P). Three different ^1^H-NMR pulse experiments were performed for each sample: 1) Nuclear Overhauser Effect Spectroscopy (NOESY)-presaturation sequence to suppress the residual water peak; 2) Carr-Purcell-Meiboom-Gill sequence (CPMG, spin-spin T2 relaxation filter) with a total time filter of 410 ms to attenuate the signals of serum macro-molecules to a residual level; 20 ppm spectral width and a total of 64 transients collected into 64 k data points, and 3) Diffusion-edited pulse sequence with bipolar gradients along with longitudinal eddy-current delay (LED) to further estimate the serum lipoprotein profile according to our recent described methodology [21].

The second aliquot was used for GC/MS analysis according to Agilent's specifications [22]. 100 µL of serum were spiked with 20 µL of internal standard solution (1 µg/µL succinic-d4 acid; Sigma-Aldrich). After protein precipitation using 900 µL of cold methanol/water (8∶1 v/v) samples were centrifuged 10 minutes at 4°C, and 200 µL of the supernatant were spiked with 20 µL of myristic acid-d27 (Sigma Aldrich) used for retention time lock. Samples were then lyophilized, and dissolved and incubated in 50 µL of methoxyamine in pyridine (0.3 µg/µL) during 16 hours at room temperature. Derivatization by silylation reagents was done using 30 µL of N-methyl-N-trimethylsilyltrifluoroacetamide with 1% trimethylchlorosilane (MSTFA+1% TMCS, Sigma) during 1 hour at room temperature. Samples were automatically injected into a GC–MS system (HP 6890 Series gas chromatograph coupled to a mass selective detector model 5973) equipped with a J&W Scientific DB 5-MS+DG stationary phase column (30 m×0.25 mm i.d., 0.1 µm film) (Agilent Technologies). The injector temperature was set at 250°C, and the helium carrier flow rate was kept constant at 1.1 mL/min. The column temperature was held at 60°C for 1 min, then increased to 325°C at a rate of 10°C/min and held at 325°C for 10 min. The detector operated in the electron impact ionization mode (70 eV) and mass spectra were recorded after a solvent delay of 4 min with 2.46 scans per second (mass scanning range of m/z 50–600; threshold abundance value of 50 counts). The source temperature and quadrupole temperature were 230 and 150°C, respectively.

The third aliquot was filtered through a 0.22 µm nylon membrane filter and directly injected in a HPLC system (1200 series, Agilent Technologies) coupled to a time-of-flight (TOF) mass spectometer (6210 Agilent Technologies) operated either in positive (ESI+) or negative (ESI−) electrospray ionization in full scan mode. Serum extractions were separated using a Kinetex C18, 2.6 µm, 150×2.1 mm, 100 A (Phenomenex, Torrance, CA) at a flow rate of 0.4 mL/min. The solvent system was: A = 0,1% formic acid in water; B = 0,1% formic acid in acetonitrile (ACN). The gradient profile started linearly from 2% to 20% buffer B in 3 min and was followed by another linear gradient from 20% to 100% buffer B in 18 min and hold for 7 min at 100% buffer B. The injection volume was 15 µL. The instrument was set to acquire over the m/z range 80–1000 with an acquisition rate of 1.3 spectra/second. MS/MS data of the metabolites of interest was collected using an HPLC-ESI QqQ system (6410, Agilent Technologies) using identical LC conditions.

Quality control samples (QCs) consisting of pooled serum samples of all patients entering the study were used. In our LC-MS platform, QCs were injected before the first study samples were analyzed and then periodically after 5-study samples. For GC-MS, QCs were injected periodically after 10-study samples. In addition, to begin with the chromatographic analysis, injection of 3 blank runs were performed both in LC/MS and GC/MS. Furthermore, samples entering the study were entirely randomized to reduce systematic error associated with instrumental drift.

### Ethics

This clinical study was registered as ISRCTN12871246 and conducted in Sant Joan de Déu University Hospital (Barcelona, Spain), without support from industry, after approval by the Institutional Review Board of Sant Joan de Déu University Hospital, and after written informed consent by each patient.

### Data analysis and statistical methods

The acquired CPMG NMR spectra were phased, baseline-corrected and referenced to the chemical shift of the α-glucose anomeric proton doublet at 5.22 ppm. Pure compound references in BBioref AMIX (Bruker); HMDB and Chenomx databases were used for metabolite identification. After baseline correction, intensities of each ^1^H-NMR regions identified in the CPMG 1D-NMR spectra were integrated for each sample entering the study using the AMIX 3.8 software package (Bruker, GmBH). To identify discriminating markers, intensities of each of the identified spectral regions in the untreated PCOS serum spectra were compared against the same spectral regions on their treated counterparts using principal component analysis (PCA) of the auto-scaled within-subject variation dataset derived from multilevel simultaneous component analysis (MSCA) [23] and the non-parametric Wilcoxon-rank summed test. LC/MS (ESI+ and ESI− mode) and GC/MS data were processed using the XCMS software [24] (version 1.6.1) to detect and align features. A feature is defined as a molecular entity with a unique m/z and a specific retention time. XCMS analysis of these data provided a matrix containing the retention time, *m/z* value, and integrated peak area of each feature for every serum sample extraction discussed above. The tab-separated text files containing GC/MS data were imported into Matlab where normalization to internal standard succinic acid-d4 was also performed. QCs were always projected in a PCA model together with the study samples to verify that technical issues do not mask biological information. Basal PCOS samples and their treated counterparts were compared using the integrated peak area of each feature via PCA of the auto-scaled within-subject variation dataset derived from MSCA and the non-parametric Wilcoxon-rank summed test, and assigning a fold value to indicate the level of differential regulation due to the 30-months treatment. Differentially regulated metabolites (fold>2) that were statistically significant (p<0,05) detected by LC/MS were characterized by MS/MS using a LC-QqQ instrument. Differentially regulated metabolites detected by GC/MS were identified using the NIST and Fiehn mass spectral libraries. In addition, the retention time of pure standards were confirmed. Data (pre-) processing, data analysis, and statistical calculations were performed in Matlab (Matlab version 6.5.1, Release 13). The MSCA matlab code was downloaded and adapted from www.bdagroup.nl.

## Results

### Low-dose Pio/Flut/Met polytherapy improves biochemical long-term health markers in PCOS patients

A series of biochemical parameters were initially monitored in PCOS patients prior to and at the end of the 30 months treatment. The results summarized in Table 1 indicate that the treatment caused a broad spectrum of biochemical adjustments, including a marked reduction in serum concentrations of androgens such as testosterone (−41±9%, p = 0.0026) and androstenedione (−35±5%; p = 0.003), whereas insignificant changes in body weight were measured. The carotid IMT was markedly reduced (−33±3%; p = 3.0×10^−5^) after the treatment, and it was accompanied by a significant augment of HDL cholesterol levels in serum (34±5%, p = 0.005). In addition, some PCOS patients decreased their visceral fat mass considerably after the treatment. Of note, markers of liver dysfunction such as transaminases and lactate dehydrogenase remained unaltered (data not shown). Overall, these changes are clinically associated with a phenotypic improvement in PCOS patients after the treatment, and complement the metabolomic analysis.

**Table 1:** Endocrine-metabolic markers, carotid IMT and abdominal fat partitioning at baseline and after 30 months of low dose Pio/Flu/Met polytherapy.

	At baseline (N = 12)	At 30 months (N = 12)	p-values
Age (yr)	19.8±0.4		-
BMI (Kg/m^2^)	22.3±1	22.5±1.1	0.9770
Score F&G	16.1±1.3	8±0.6	0.0001
Total cholesterol (mg/dL)	165.3±7.1	185±8.1	0.0606
HDL-cholesterol (mg/dL)	50.8±2.2	68.4±4.2	0.0055
LDL-cholesterol (mg/dL)	102.3±6.1	96.9±5.4	0.7726
Triglycerides (mg/dL)	64.4±5.3	97.7±9.7	0.0093
Testosterone (ng/dL)	77.2±6	43.9±6.6	0.0026
SHBG (nmol/L)	37.8±3.8	164.3±8.6	4.0×10^−5^
FAI	8.5±1.8	0.9±0.2	4.0×10^−5^
Androstendione (ng/dL)	444.3±47	276±25	0.0030
DHEAS (µg/dL)	265±37	202±33	0.2365
Carotid IMT (mm)	0.46±0.02	0.31±0.01	3.0×10^−5^
Subcutaneus (Sc) fat mass (cm^2^)	144±50	151±23	0.8852
Visceral (Vis) fat mass (cm^2^)	68±17	40±8	0.0783
Vis/Sc fat mass	0.39±0.04	0.20±0.04	0.1484

Values are mean ± SEM; BMI, body mass index; SHBG, sex hormone-binding globulin; DHEAS, dehydroepiandrosterone-sulfate; FAI, free androgen index; IMT, Intima-Media Thickness.

### Multivariate Data Analysis of PCOS Serum Samples

**Figure 1:**
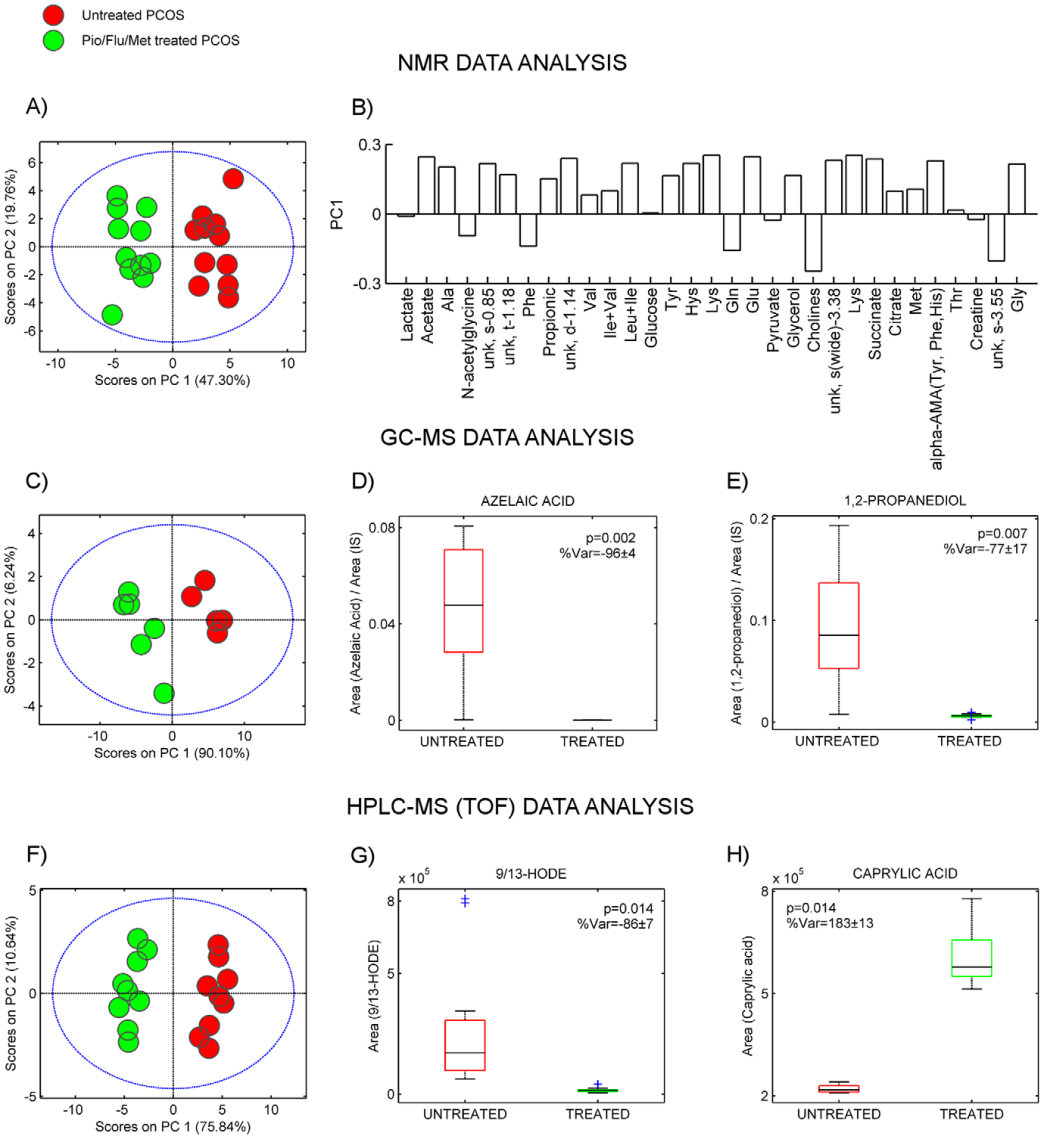
Multivariate modelling of ^1^N-NMR, GC/MS and LC/ESI-TOF MS data. (**A**) PC1/PC2 scatter scores plot and (**B**) PC1 loading bar plot of PCA calculated on the within-subject matrix derived from the MSCA modelling of the 32 selected spectral regions identified in the ^1^H-NMR CPMG serum spectra of untreated (red markers) and treated (green markers) PCOS patients. (**C**) PC1/PC2 scatter scores plot of PCA calculated on the within-subject matrix derived from the MSCA modelling of GC/MS data. Boxplots of (**D**) azelaic acid and (**E**) 1,2-proanediol, the two metabolites corresponding to the most discriminating features along the corresponding PC1 loadings bar plot. (**F**) PC1/PC2 scatter scores plot of PCA calculated on the within-subject matrix derived from the MSCA modelling of LC/ESI-TOF MS data. Boxplots of (**G**) 9- and 13-HODE, and (**H**) caprylic acid, the two metabolites corresponding to the most discriminating features along the PC1 loadings bar plot. P-values derived from Wilcoxon rank-summed pair-matched comparison of untreated and treated PCOS patients. Mean ± sem of the percentage of variation are also indicated.

Given the longitudinal nature of our study, we used a multilevel simultaneous component analysis (MSCA) method [23] to examine independently different types of variation in the NMR and MS-based metabolomic data, namely, variation between patients and variation in time within patients as a result of the polytherapy. MSCA enables to split the variation within-subjects that accounts for the variation before and after the treatment for each patient, from the variation between-subjects that accounts for the biological variation (i.e., genotype differences) in two different data matrices. The variation within-subjects is represented in the PCA plot of Figure 1. The PC1/PC2 scores plot of the ^1^H-NMR CPMG spin-echo experiment shown in Figure 1A reveals a clear clustering along PC1 (∼57% of the variance), which accounts for the post-treatment variation of each patient. Figure 1B depicts the bar loading plot for PC1, the higher the absolute bar value the higher the influence of such variable in the variation induced by the treatment. Hence, PCOS serum samples at the end of the treatment were characterized by rather larger values of choline-containing molecules and diminished values of 1,2-propanediol and lysine, among others. Similar PC1/PC2 scores plot was observed with the GC/MS and LC/MS derived data (Figure 1C and Figure 1F), confirming a profound metabolic variation within patients due to the 30 months polytherapy. Loadings bar plot were also studied and some of the features implicated in this variation were depicted as boxplots in Figures 1D–H. Altogether our MSCA model set apart the biological variation between patients and highlighted within-patient variation due to the Pio/Flu/Met polytherapy, showing clear differences in the relative concentration of specific compounds in the serum of PCOS patients. Accordingly, we focused in the characterization of the most discriminating compounds of the MSCA model.

### Analysis of serum ^1^H-NMR spectra

**Figure 2:**
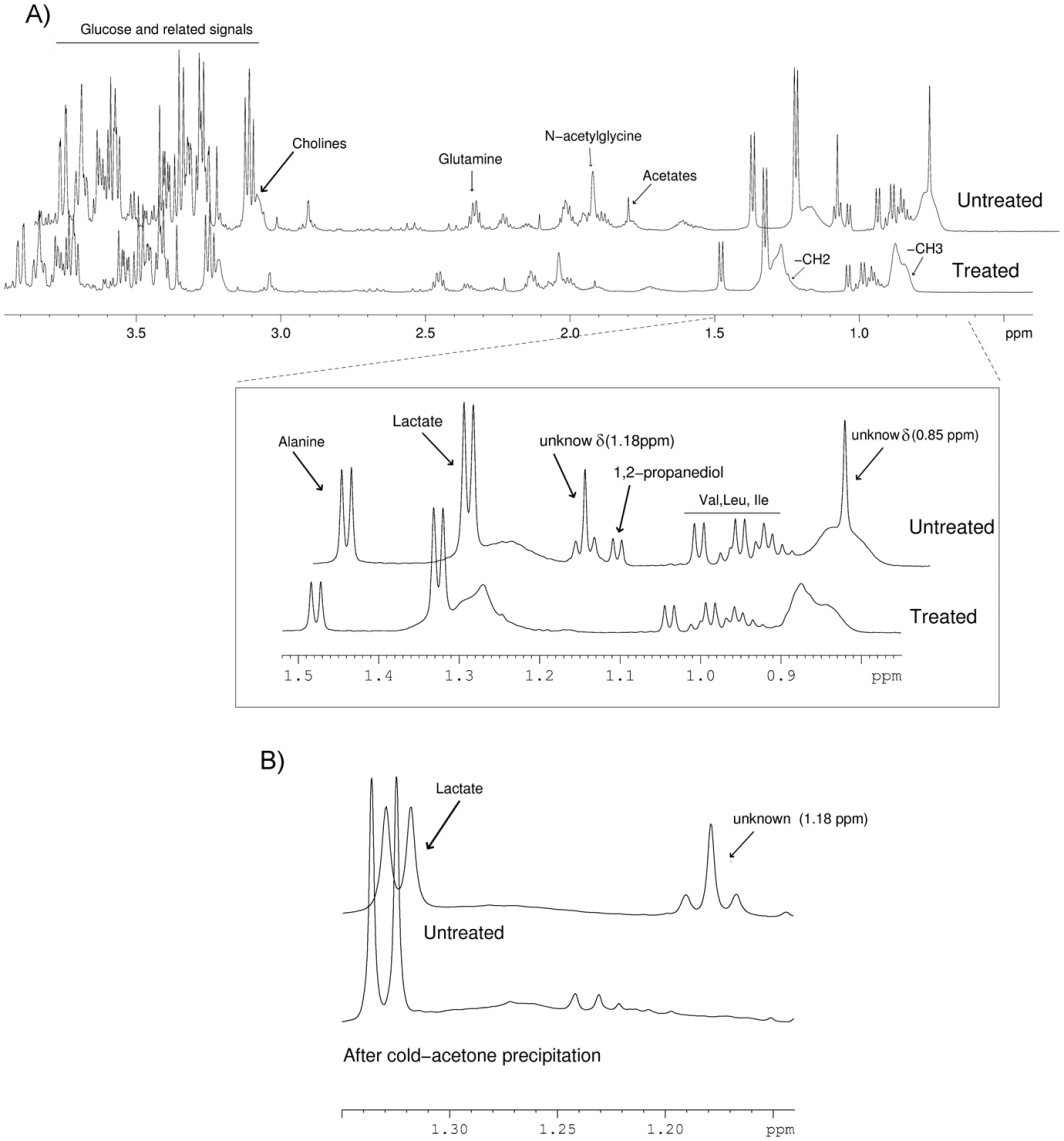
CPMG ^1^H NMR spectra of a representative PCOS patient's serum. (**A**) Comparative spectra at baseline and after 30 months of Pio/Flu/Met polytherapy. CPMG spin echo experiment allows filtering broad signals of lipid and lipoproteins enhancing low-molecular weight metabolites such as amino acids, lactate and intermediate metabolites. The inset displays an expanded δ (0.75–1.5 ppm) spectral region showing two unidentified resonances characteristic of the serum spectra of untreated PCOS patients: a broad singlet arising at 0.85 ppm and three peaks centered at 1.18 ppm. (**B**) CPMG ^1^H-NMR spectra of the same untreated PCOS patient shown in Figure 2A before and after cold acetone precipitation. After acetone precipitation the three signals centered at 1.18 ppm were depleted, confirming the occurrence of oxidized lipoprotein-related structures in the serum of PCOS women.

The CPMG ^1^H NMR serum spectra are composed of overlapped resonances from low molecular weight metabolites such as amino acids or lactate and T_2_ lipoprotein attenuated signals. Figure 2A depicts representative CPMG ^1^H NMR spectra of a PCOS patient's serum prior to and at the end to the 30 months Pio/Flu/Met polytherapy. Clear differences can be observed between the same serum sample prior to and after the 30 months treatment. The polytherapy induced lipoprotein rearrangements reflected in resonances attributable to both methylene (δ 1.25 ppm) and methyl (δ 0.85 ppm) terminal groups of fatty acids contained in LDL and VLDL particles. In contrast, a depletion of the 1,2-propanediol doublet at 1.14 ppm and the acetate broad singlet arising at 1.91 ppm was observed after the treatment. Also, two prominent unidentified resonances arisen in the serum spectra of PCOS patients prior to the treatment were decreased as a result of the polytherapy. The first resonance corresponded to a broad singlet (multiplicity examined by 2D J-res) at 0.85 ppm, and the second unknown resonance was composed of three different peaks centered at 1.18 ppm which probably do not correspond with a triplet signal because their intensities did not match exactly with the established 1∶2∶1 triplet intensity ratio.

Table 2 summarizes the statistical values, detailed moieties assignments and structural identities of the metabolites analyzed using CPMG ^1^H NMR. The relative concentration of glutamate, 1,2-propanediol, lysine, and succinate in serum samples was markedly decreased as a result of the treatment. In contrast, NMR signals attributed to N-(CH_3_)_3_ groups of choline-containing molecules were increased after the treatment. In addition, the three unknown NMR signals centered at 1.18 ppm, which match the characteristic pattern of resonances found in the serum spectra of patients with coronary heart disease (CHD) reported by Jankowski et al. [25], decreased in the spectra after the treatment. Such specific pattern of resonances corresponded to oxidized LDL particles, as demonstrated by Jankowski and co-workers when they compared the spectrum of lipoprotein subfractions of patients with CHD and LDL particles oxidized *in vitro* using Cu^2+^. To confirm the association of lipoprotein particles with these signals we precipitated serum proteins from untreated patients using cold-acetone and recorded the ^1^H-NMR spectra on the supernatant. As showed in Figure 2B, the three signals centered at 1.18 ppm disappeared after cold acetone precipitation, suggesting an association with lipoprotein-related particles. Overall, our results suggest that the Pio/Flu/Met polytherapy has a dramatic effect on the lipoprotein profile of PCOS patients.

**Table 2:** Summary of the metabolites found to be significantly varied in either analytical platform after 30 months low-dose Pio-Flu-Met polytherapy.

^1^H-NMR analysis (N = 12)						
Metabolite	δ(ppm)	multiplicity	p-values	Mean (%Variation) ±SEM	Moieties assignments	Comments
unknown*	0.85	s (broad)	0.0038	−35±19	-	*oxLDL related structures
1,2-propanediol	1.14	d (6.5 Hz)	0.0010	−65±27	2(-CH_3_-)	
unknown*	1.18	3×s	0.0097	−24±27	-	*oxLDL related structures
Lysine	1.69	m	0.0008	−45±4	δ-CH_2_	
Acetates	1.91	s	0.0008	−55±6	CH_3_	
Glutamate	2.35	m	0.0008	−54±4	γ-CH_2_	
Succinate	1.76	s	0.0008	−42±5	2 (-CH_2_-)	
Cholines-containing molecules	3.21	s	0.0149	120±22	N-(CH_3_)_3_	
unknown	3.35	s	0.0010	156±41	-	
unknown	3.38	s (broad)	0.0010	−35±5	-	
unknown*	3.65	m	0.0074	−34±10	-	*oxLDL related structures

GC-MS analysis (N = 6)					
Metabolite	m/z ion	RT(min)	p-values	Mean (%Variation) ±SEM	EI-confirmatory m/z ions
1,2-propanediol§	117	6.05	0.0069	−77±17	-
Nonanoic acid	215	11.15	0.0069	−68±7	-
Glutaric acid§	261	11.75	0.0421	−91±6	-
Glutamate	186	13.37	0.0037	−58±9	56,84
Azelaic acid§	317	16.38	0.0021	−96±4	124,152,125,201,199,107,317,129,318,183,111,204,75,97,153,55,67,185,197,81,123,171

LC/ESI-TOF MS analysis (N = 9)								
Metabolite	[M-H]^−^	RT(min)	p-values	Mean (%Variation) ±SEM	[M]	Formula	δ(ppm)	MS/MS diagnostic ions
Caprylic acid	143.1068	23.2	0.0143	183±13	144.1141	C8H16O2	6	143@20eV
9/13-HODE	295.2282	8.7	0.0143	−86±7	296.2357	C18H32O3	1	295,277,195@20eV

d = doublet, s = singlet, m = multiplet. Percentage of variation was calculated for each patient as the area of the spectral region or selected XCMS feature at baseline minus the area of the same feature or spectral region after the treatment relative to the former. Values are expressed as mean ± SEM. A negative value indicates that levels of the corresponding metabolite resulted significantly decreased with the treatment while positive values indicate a significant increase. p-values correspond to Wilcoxon rank-summed test and FDR correction. Statistical significance was considered for those spectral regions or features having p-corrected values<0.05 and fold changes>2;

§ Indicates those metabolites whose retention time and mass spectra were checked using pure standard references.

### Lipoprotein rearrangements after low-dose Pio/Flu/Met intervention

**Figure 3:**
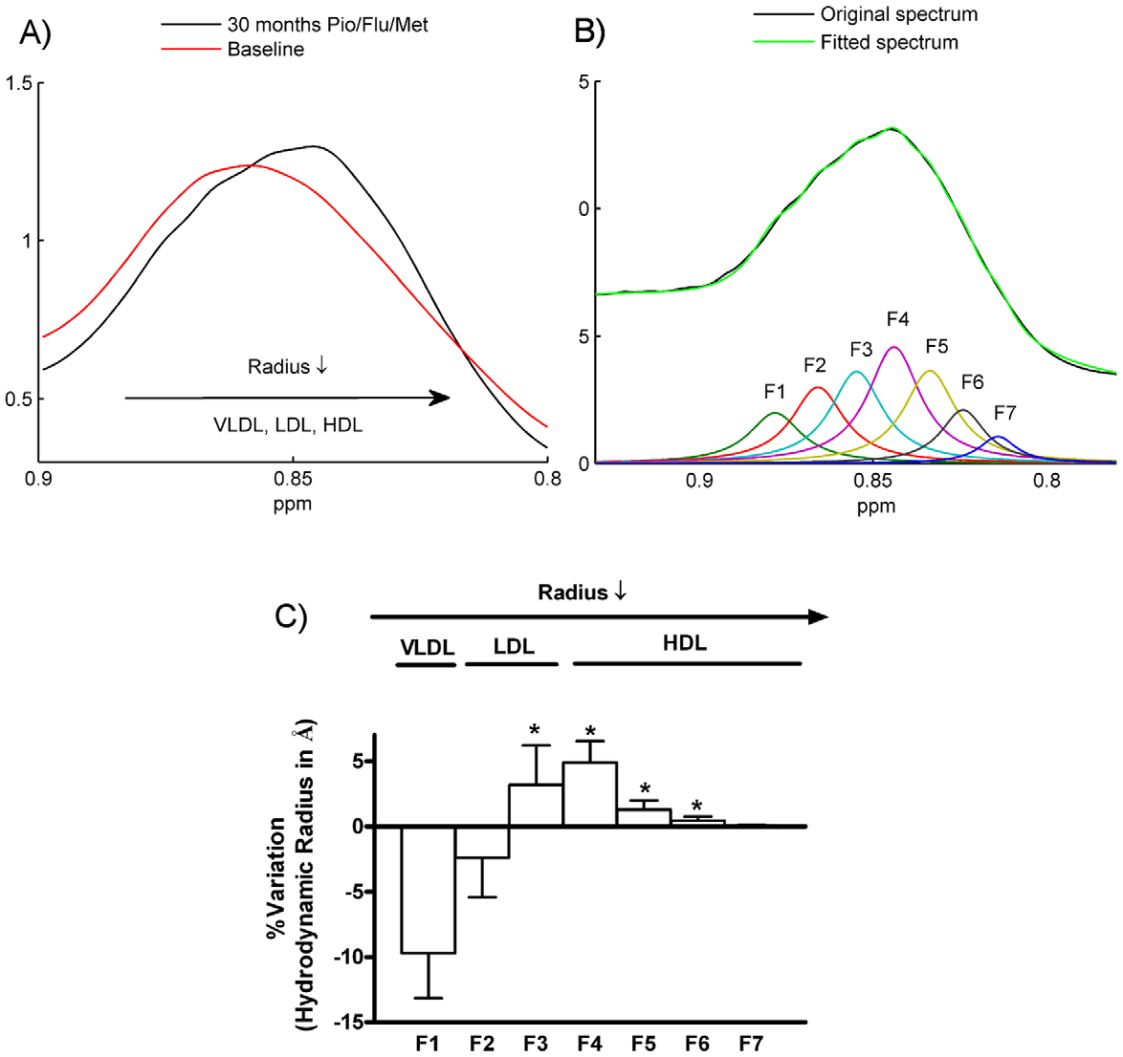
Lipoprotein ^1^H-NMR analysis. (**A**) Comparative bipolar-LED diffusion mean spectra of the methyl region (δ 0.85 ppm) for untreated and treated PCOS patients. (**B**) Bipolar LED pulse sequence ^1^H NMR spectra of a treated PCOS serum showing the fitting of the methyl band using the seven Lorentzian functions derived from our previously described methodology. Black line represents the original methyl envelope and green line the reconstructed spectrum after the fitting. (**C**) The estimated radiuses of lipoprotein particles in serum calculated using the seven Lorentzian functions were compared at baseline and after 30 months of Pio/Flu/Met polytherapy.

To confirm that the lipoprotein profile is severely affected by the Pio/Flu/Met treatment, we measured the size of lipoprotein particles in serum samples prior to and at the end of the 30 months intervention, according to our recently described methodology [21]. In brief, the size of different lipoprotein subclasses were estimated using up to seven Lorentzian functions to fit the methyl peak surface obtained from 2D (bipolar-LED) diffusion edited ^1^H-NMR experiments. Then, diffusion coefficients and hydrodynamic radius through Stokes-Einstein equation for each one of these seven functions were estimated. Figure 3A shows the average methyl spectrum centered at 0.85 ppm after 30 months of polytherapy in relation to the average signal at baseline. The shift to the upfield region in the spectra after the treatment indicates a greater contribution of the HDL subfraction to the signal of methyl groups. Figure 3B shows the fitted spectrum of a treated PCOS patient using the seven Lorentzian functions. Based on the previously measured diffusion coefficients described in our methodology [21], we estimated the hydrodynamic radius (i.e., size) for each of these functions. Figure 3C shows the mean percentage of variation of the estimated radius for each function as a result of the Pio/Flu/Met treatment. The polytherapy resulted in significantly increased radiuses associated with atherogenic small, dense LDL (F3) and protective large HDL (F4–F6) lipoprotein subclasses.

### Mass spectrometry analysis

**Figure 4:**
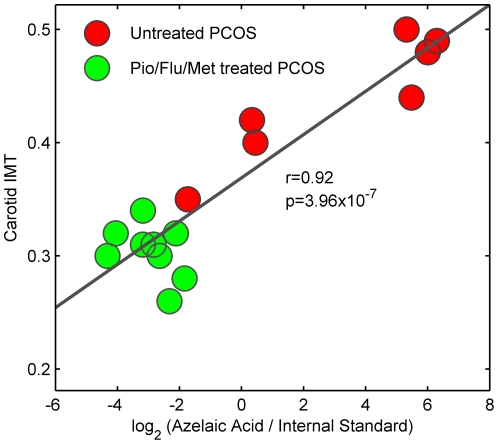
Correlation between IMT and azelaic acid levels in serum. Positive significant correlation (r = 0.92, p = 3.96×10^−7^) between carotid IMT values and azelaic acid levels. Azelaic acid levels were calculated as the ratio of the fragmentation peak of azelaic acid at m/z = 317 (retention time = 16.38 min) and the peak area of the internal standard. Red and green dots represent values of untreated and treated PCOS patients respectively.

Our mass spectrometry-based platform involves LC-ESI-TOF-MS and GC-single quad MS profiling followed by data analysis with the open-source software XCMS. The relative abundance of metabolites in serum samples was quantified by comparing the integrated area of each feature, and calculating the percentage of variation of such feature to indicate the level of differential regulation prior to and at the end of the Pio/Flu/Met intervention. Some of the most up-regulated metabolites were identified by tandem MS/MS. Metabolite annotations and statistical analysis are summarized in Table 2. Metabolites identified using GC/MS were consistent with NMR data. For example, the levels of glutamate and 1,2-propanediol in serum were also significantly decreased after the polytherapy. In addition, the treatment led to decreased levels of nonanoic, glutaric and azelaic acid. Of note, the level of azelaic acid resulted positively correlated with carotid IMT (r = 0.92, p = 3.96×10^−7^) (Figure 4). LC/MS data showed that the treatment resulted in significantly increased level of caprylic acid, whereas it induced a marked reduction in the level of 9-HODE and 13-HODE, the most abundant mono-hydroxyderivative forms resulting from the oxidation of linoleic acid.

## Discussion

**Figure 5:**
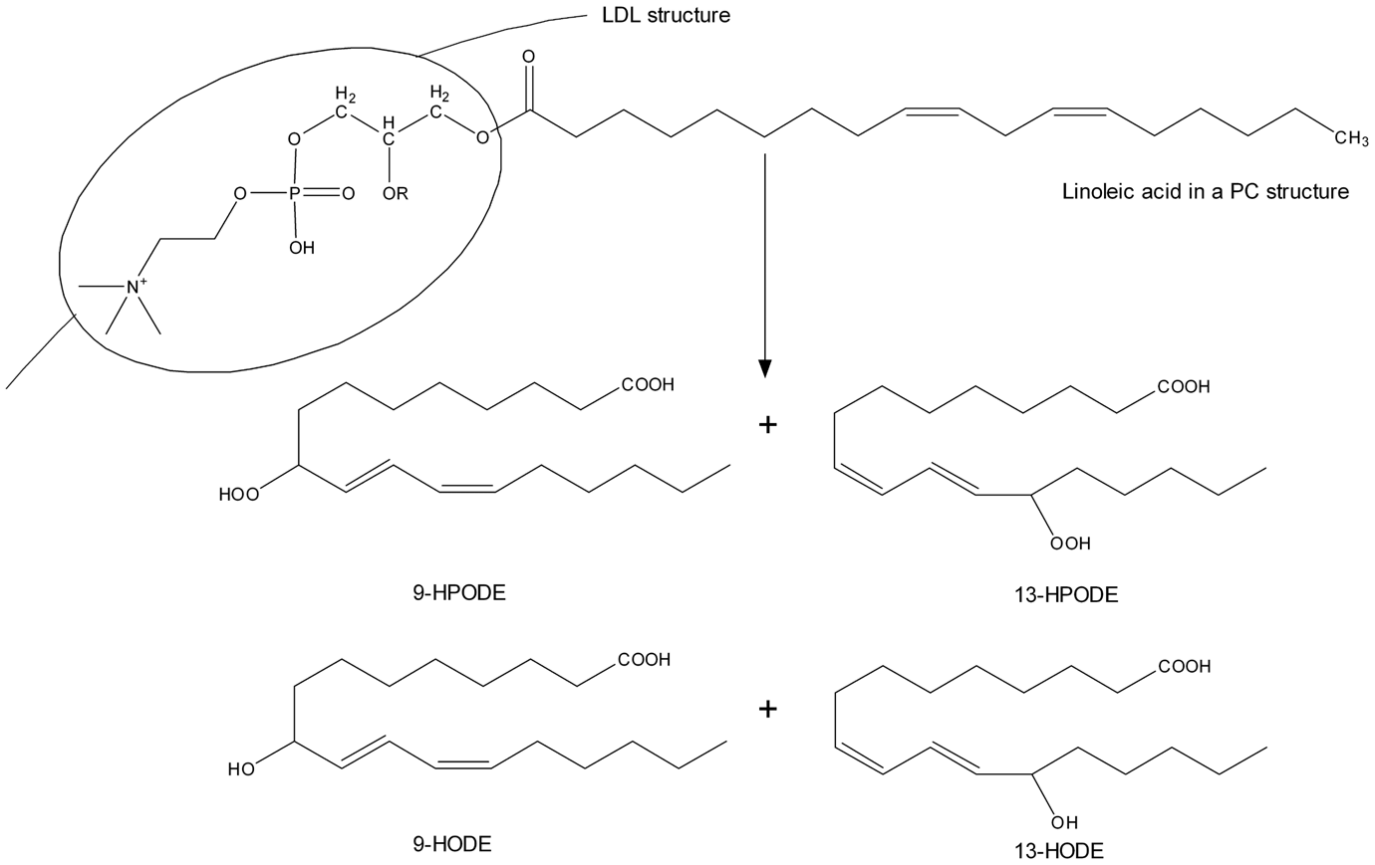
Linoleic acid oxidation products. Formation of linoleic acid hydroperoxyderivatives (HPODEs) are further reduced to their corresponding hydroxyderivatives (HODES). Chemical structures of linoleic acid (18∶2); 9- and 13-hydroperoxylinoleic acid (9- and 13-HPODE); and 9 and 13-hydroxylinoleic acid (9- and 13-HODE).

The underlying causes of PCOS are still unknown, and therefore the medical treatment is tailored to the patient's symptoms. Typically, these are lowering insulin level, restoration of fertility and regular menstruation, and treatment of hirsutism and acne. General interventions such as low-dose flutamide, metformin and pioglitazone in combination with an estro-progestagen can be very beneficial because it confers further improvements of the endocrine-metabolic state. Our metabolomic analysis has revealed that the polytherapy induces an increase in the estimated radius of small, dense LDL lipoprotein subclasses together with a reduction in the level of oxidized LDL particles. These macromolecular rearrangements are associated with changes in the level of specific downstream metabolites produced by linoleic acid peroxidation (i.e. 9-HODE, 13-HODE). Linoleic acid represents the most abundant polyunsaturated fatty acid in LDL particles. Oxidation of LDL transforms linoleic acid into different hydroperoxyderivative (HPODEs) isomers [26], which are subsequently reduced and released by specific lipases from the membrane lipids as free hydroxyoctadecadienoic acid (HODE), such as 9- and 13-HODE, identified in our study [27] (Figure 5). In addition, the oxidative modification and degradation of fatty acids contained in LDL particles generates a complex array of shorter chain-length fragments that covalently modify ε-amino groups of lysine residues of the protein moiety to generate the oxidatively modified LDL particles [28], [29], [30]. Among these shorter chain-length fragments causing structural modifications of proteins, we have identified azelaic acid and glutaric acid [31]. It is worth mentioning that azelaoyl phosphatidylcholine (azPC) accounts for almost two-thirds of the oxidized phospholipids in oxLDL [32], [33]. Also, accumulation of azelaic acid and glutaric acid in plasma of diabetic rat models and type 1 diabetic patients, respectively, has been reported previously [34], Overall, our NMR- and MS-based metabolomics study demonstrate that the Pio/Flu/Met polytherapy reduces the amount of oxidized lipoprotein particles and downstream oxidative metabolites in the serum of PCOS patients.

Previous studies explored the effect of flutamide, metformin, and pioglitazone in monotherapy or in combination therapy. For example, combined Pio/Met therapy in type 2 diabetic patients improved their specific lipid abnormalities [35], [36], [37], [38]. Pioglitazone, when used both in monotherapy or in combination therapy, modifies the atherogenic lipoprotein profile reducing triglycerides, increasing the larger HDL_2_ subfractions and improving the HDL cholesterol load [39]. Very few studies, however, have addressed in detail the effects of pioglitazone and metformin in LDL subfractions. Lawrence et al [40] reported a significant fall in LDL_3_ mass and LDL_3_ proportion in overweight type 2 diabetic patients treated with metformin alone. The total cholesterol–to–apoB ratio (used as a surrogate marker for changes in LDL subfraction distribution), however, remained unchanged. In contrast, when overweight type 2 diabetic patients were treated with pioglitazone alone, a significant increase in the cholesterol-to-apoB ratio was reported, indicating larger (and potentially less atherogenic) LDL particles. Pioglitazone alone also induced an increase of LDL particles diameter and a decrease in LDL density in normolipidemic, nondiabetic patients with hypertension [41]. Finally, the treatment of Goto-Kakizaki rats (a type 2 diabetes model) with pioglitazone reduced the levels of lipid peroxides in plasma and the susceptibility of LDL particles to oxidation [42]. Our findings result in good agreement with previous studies and evidence that the addition of pioglitazone to the combined flutamide/metformin polytherapy induces an increase in the mean diameter particles of small LDL subfractions. Besides, our comprehensive metabolomic approach allowed us not only to detect changes in the size of the different lipoprotein particles but also in downstream, oxidation products such as 9- and 13-HODE, azelaic acid and glutaric acid. Overall, our results demonstrate the utility of metabolomics to explore the effect of medical treatments on metabolic alterations. In this study, the combined pioglitazone/flutamide/metformin polytherapy reverses the oxidant status of untreated PCOS patients. Given that oxidation of LDL particles has been suggested to be the key triggering event in the progression of atherosclerotic lesions [43], and that the carotid IMT is also markedly reduced after 30 months of polytherapy, we postulate that untreated young PCOS women may suffer early stages of atherosclerosis that could potentially have deleterious effects at older ages. Besides, we have demonstrated that azelaic acid levels are strongly correlated with carotid IMT. Hence we suggest that azelaic acid can be considered as an early marker of lipoprotein oxidation and subclinical atherosclerosis.
